# Different yawns, different functions? Testing social hypotheses on spontaneous yawning in *Theropithecus gelada*

**DOI:** 10.1038/srep04010

**Published:** 2014-02-06

**Authors:** Alessia Leone, Pier Francesco Ferrari, Elisabetta Palagi

**Affiliations:** 1 Dipartimento di Neuroscienze, Università di Parma, Parma, Italy; 2 Centro Ateneo Museo di Storia Naturale, Calci, Università di Pisa, Pisa, Italy; 3 Unità di Primatologia Cognitiva, Istituto di Scienze e Tecnologie della Cognizione, Consiglio Nazionale delle Ricerche, Roma, Italy

## Abstract

Here, we tested hypotheses about the potential functions of yawning based on its intensity and social contexts. Due to their spectrum intensity of yawns (covered teeth/YW1; uncovered teeth/YW2; uncovered gums/YW3), geladas are a good model species for this purpose. We suggest that yawns of different intensity can bear different information according to the performer, the context and the behavioural pattern temporally associated to the yawn event. YW3, mainly performed by high ranking males during periods of high social tension, was frequently associated with an auditory component and often accompanied by scratching (a measure of anxiety). YW1 and YW2, preferentially performed by females, were frequently associated to lip smacking, an affiliative display. In conclusion, even though a clear-cut functional distinction of geladas' yawn intensity is difficult, YW1 and YW2 seem to be more linked to affiliative social interactions; whereas, YW3 seems to be more linked to agonistic and tension situations.

Yawning is an involuntary and stereotyped behaviour observed in most vertebrate species (including humans) from foetal stages to adulthood^1^. In mammals, the yawning patterns include mouth opening, deep inspiration, brief apnea, and slow expiration^2^. Due to the old phylogenetic origins of this behaviour^3^, it has been proposed that it is adaptive and provides some evolutionary advantages. Recently, Guggisberg et al.^2^ suggested that due to its ubiquity across different taxa and occurrence under a number of different physiological states and social contexts, yawning may serve more than one function. For example, yawning may be involved in homeostatic processes^1^,^3^,^4^,^5^, and it may be linked to changes of environmental conditions^6^,^7^,^8^,^9^, or even to social contexts (e.g. signal of aggressiveness, hierarchical dominance, frustration, sexual excitement, or a means of synchronising activities within the group)^2^,^10^,^11^,^12^,^13^,^14^,^15^,^16^.

In human and nonhuman primates, two different types of yawn are generally distinguished according to the physiological state and social context: true/rest yawns^17^ and tension/aggressive yawns^18^. True yawns are typically associated with states of drowsiness and relaxation (sleepiness^7^,^9^ or boredom^19^). On the other hand, tension or aggressive yawns occur in conflict situations and may indicate arousal^10^,^11^,^20^,^21^,^22^. In contrast to this dichotomous view, Altmann^23^ suggested that both types of yawns may indicate levels of physiological arousal and, therefore, it is extremely difficult to disentangle the two based on the stimuli/context triggering them. For example, macaques are known to exhibit ‘emotion yawns' or ‘social yawns' during antagonistic social encounters^10^,^24^,^25^. Among the great apes, chimpanzees yawn mostly in response to human proximity^26^ and during conditions of social tension^27^. Other researchers have also proposed that in primates spontaneous yawning is a form of self-directed behaviour associated to anxiety states^21^,^28^,^29^.

Several of the reports here described seem to support the hypothesis that one of the functions of yawn is that of stimulating or facilitating arousal during state changes^1^,^17^,^30^,^31^. Although the concluding evidence supporting such hypothesis still requires further investigation, there is a general consensus on that fact that yawning is often anticipatory of important events and is associated to behavioural transitions, including sleep/awake cycles^18^,^32^,^33^,^34^,^35^.

One of the most intriguing issue related to the yawning phenomenon concerns the analysis of its behavioural patterns, which could be potentially provide critical information in order to understand its functional roles. Recently, Vick and Paukner^14^, based on a detailed behavioural analysis, identified in chimpanzees two distinct forms of yawn, a full yawn and a yawn in which the mouth is half-closed. This finding has been interpreted within the context of the possible underlying neurological mechanism. In fact, the possibility to partially regulate its expression suggests that there is a voluntary control, or at least in part, over oro-facial movements accompanying the yawn. Moreover, it is possible that these two different forms of yawn reflect different functions.

Geladas, an Old World monkey species, perform yawning at three different levels of intensity that have been recently categorized on the basis of the degree of mouth opening and the possible co-presence of vocalizations: yawn with covered teeth (YW1), yawn with uncovered teeth (YW2), and yawn with uncovered gums and head movements (YW3)^13^. Moreover, vocalizations can be associated to yawns both before and during the performance^13^. The variability in the expression of this behaviour in geladas, makes this species suitable to investigate in details the possible functional role of yawning. We therefore focussed our investigation on this species and tested specific *social hypotheses* (not mutually exclusive) on the function of yawning according to its intensity and contexts. The gelada is a good model species also because it has a strong sexual dimorphism^28^, a clear-cut linear hierarchy^36^, high social cohesiveness^37^ and fine-tuning towards companions^38^. Moreover, yawn contagion has been recently demonstrated in this species, thus suggesting its key role in social behaviour^13^. In the present study we will test three social hypotheses.

## Hypothesis 1 (sleep-awake transition)

Yawning is often associated with particular patterns of rest-activity, indicating the possibility of an endogenous temporal rhythm^1^,^8^,^10^. These internal rhythms allow individuals to anticipate and to prepare them to the environmental transitions, as well as to trigger behavioural and physiological changes in accordance to such transitions^39^. In laboratory rats, a light-to-dark transition was found to be associated with daily peaks of yawning^40^, thus suggesting the presence of a circadian rhythm. In humans, both the transition from light-to-dark and dark-to-light are associated with peak frequencies of yawn^6^,^7^, even though it seems to be more frequent after waking than before sleeping^7^. Up to now, no study explored the daily variation of the different types of yawns performed^13^. If yawning in geladas is linked to sleep/awake transition, we expect to find peak levels of this behaviour in the early morning and late evening (Prediction 1). If the yawns of different intensity respond differently to this transition we will be able to distinguish what in literature are described as true yawns from other types of yawn^10^,^11^,^14^,^17^,^23^.

## Hypothesis 2 (social display)

Threat yawns have been described in some primate species^41^,^42^, but if canine displays during yawning can be considered as an aggressive signal, remains still controversial^10^,^43^. Furthermore, “threat yawns” are fundamentally different from other types of yawns (true/relax) in that they are assumed to occur in specific social contexts (i.e. during conflicts) and to be displayed by specific subjects, such as high rank individuals^44^ directing their aggressive towards lower-rank individuals. Moreover, in highly dimorphic species (*Cercocebus albigena* and *Macaca fascicularis*^10^; *M. nigra*^11^; *M. fuscata*^12^), it is expected that it is displayed mostly by males due to their active role in territorial defence and females' control^28^. This sex difference in yawn frequency seems, in fact, to disappear in species not characterized by a pronounced dimorphism in canine size, such as humans^45^ and lemurs (*Lemur catta*, *Propithecus verreauxi*, Palagi unpublished data).

If yawning in geladas, especially in its more intense version (YW3), has a role in threatening, we expect that sex and rank has an influence on the yawning frequency (Prediction 2a). More specifically, we expect YW3 to be more frequently displayed during contexts of high social tension, such as during agonistic and competitive interactions (Prediction 2b), which in geladas are mainly up to alpha males. Moreover, we expect that YW3 is frequently enriched by an auditory component (multimodal signal) which makes the behaviour less ambiguous and more easily detectable by all group members and potential rivals belonging to other OMUs (Prediction 2c). Conversely, if YW1 and YW2 are linked to a relaxed predisposition to interact socially and positively as suggested in recent reports^13^, we expect that these two patterns of yawns are mainly performed by high ranking subjects, both males and females, as a form of appeasement (Prediction 2d), and that they are temporally associated to reassuring signals such as lip smacking, which is frequently performed along with affiliative and parental care behaviours (e.g., grooming, body contact, play, lactating)^36^,^46^,^47^,^48^ (Prediction 2e).

## Hypothesis 3 (general arousal)

As self-scratching is a reliable indicator of arousal in primates^21^,^27^,^29^,^49^,^50^, the association between higher rates of scratching and yawns may be indicative of increased physiological arousal^14^,^27^,^51^. If yawning in geladas is linked to a change in motivational internal state of the performer we expect an increase in scratching levels immediately after a yawning event. More specifically, if the three types of yawn are characterized by differences in intensity and not in quality, we expect that YW3 (full yawn) is indicative of a higher variation in arousal compared to the other types (YW1 and YW2). Moreover, we expect that scratching frequency is maximum following YW3 (Prediction 3).

## Results

We collected 5,909 bouts of yawning (YW) during four years of data collection on the colony of geladas hosted at NaturZoo Rheine (see Table 1 for group composition).

Adult (mean hourly frequency ± SE: YW1 = 0.08 ± 0.010; YW2 = 0.07 ± 0.009; YW3 = 0.113 ± 0.053) yawned much more frequently than immature subjects (YW1 = 0.01 ± 0.002; YW2 = 0.053 ± 0.051; YW3 = 0.00 ± 0.00) (YW1, Mann Whitney U = 4.5; N_imm_ = 9; N_ad_ = 29; p = 0.0001; YW2, U = 13.5; N_imm_ = 9; N_ad_ = 29; p = 0.0001; YW3, U = 29.0; N_imm_ = 9; N_ad_ = 29; p = 0.0001). For this reason we restricted the analysis to adults.

As already reported in Palagi et al.^13^, we recorded yawns according to their spectrum of intensity: covered teeth (YW1), uncovered teeth (YW2), and uncovered gums (YW3).

**Figure 1:**
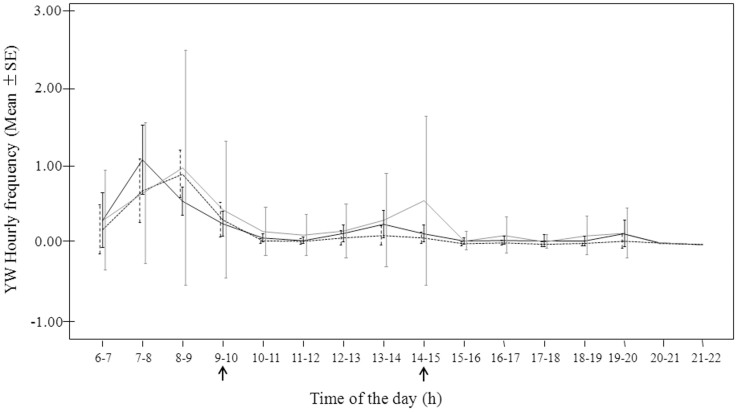
Daily hourly frequency (Mean ± SE) distribution of the three types of yawning (YW1: black line; YW2: dotted black line; YW3: grey line). The black arrows indicate the prefeeding and feeding periods. Post-hoc analysis (Dunnett's test), only statistical differences are reported: (YW1_6–7_ *vs* YW1_7–8_: q = 7.23, p < 0.001; YW1_8–9_ vs YW1_9–10_: q = 8.16, p < 0.001; YW2_7–8_ *vs* YW2_8–9_: q = 10.59, p < 0.001; YW2_8–9_ *vs* YW2_9–10_: q = 7.82, p < 0.001; YW3_7–8_ *vs* YW3_8–9_: q = 9.01; p < 0.001; YW3_8–9_ *vs* YW3_9–10_: q = 9.00; p < 0.001; YW3_13–14_ *vs* YW3_14–15_: q = 3.46, p < 0.01; YW3_14–15_ *vs* YW3_15–16_: q = 2.87; p < 0.01).

Each type of spontaneous yawning peaked in the early morning (07.00–09.00 am) (Y1: Friedman's χ^2^ = 65.938; n = 10; df = 15; p = 0.0001; Y2: Friedman's χ^2^ = 65.538; n = 10; df = 15; p = 0.0001; Y3: Friedman's χ^2^ = 75.407; n = 10; df = 15; p = 0.0001). YW1 peaked in the 07.00–08.00 time window, while YW2 and YW3 peaked slightly later in time (08.00–09.00 am) when animals were always all clumped together but completely awake. Moreover, YW3 peaked during the 2.00–3-00 pm time window, which included the afternoon prefeeding (2.00–2.30 pm). The daily time course of the different types of spontaneous yawns (YW1, YW2, YW3) is described in Figure 1 (Prediction 1 partially supported) (for the results of post-hoc tests see the caption of Fig. 1).

**Figure 2:**
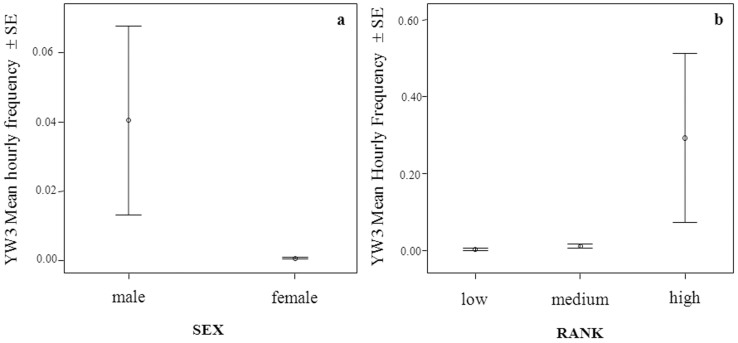
Mean (±SE) hourly frequency of YW3 as a function of sex (a) and of the ranking position of the adult subjects (b). For *p* values see Table 3.

Via LMM we evaluated the influence of sex and rank on the frequency of YW1, YW2, and YW3, separately (Table 2 and 3). The individual hourly frequency of each type of yawn (YW1, YW2, and YW3) was entered as dependent variable (Table 2). Rank and sex remained in the best model for each type of yawn (YW1; AICc = −142.76; YW2; AICc = −160.55; YW3; AICc = −11.43). For YW1 and YW2, only rank had a significant impact (YW1_rank_, F = 8.235, df1 = 2, df2 = 49.520, P = 0.001; YW1_sex_, F = 0.023, df1 = 1, df2 = 49.628, P = 0.879; YW2_rank_, F = 6.281, df1 = 2, df2 = 33.991, P = 0.005; YW2_sex_, F = 3.553, df1 = 1, df2 = 30.778, P = 0.690) (Prediction 2 d supported). For YW3, both sex and rank were significant (YW3_sex_, F = 21.751, df1 = 1, df2 = 51.807, P = 0.0001; YW3_rank_, F = 3.793, df1 = 2, df2 = 51.670, P = 0.029; Fig. 2). YW3 was more frequent in males than in females (Mann Whitney U = 6.5; N_males_ = 9; N_females_ = 20; p = 0.0001) (Prediction 2 a supported).

We also analyzed the distribution of the three types of yawns within each sex class obtaining the following results. The distribution of female YW1, YW2, and YW3 significantly differed (Friedman's χ2 = 23.227, n = 20, df = 2, p = 0.0001). In females, YW1 and YW2 were more frequent than YW3 (post-hoc Dunnett's test; YW1 vs YW2, q = 1.49, n.s.; YW1 vs YW3, q = 6.86, p < 0.01; YW2 vs YW3, q = 5.90, p < 0.01). The distribution of male YW1, YW2, and YW3 did not significantly differ (Friedman's χ^2^ = 4.333, n = 9, df = 2, p = 0.142).

**Figure 3:**
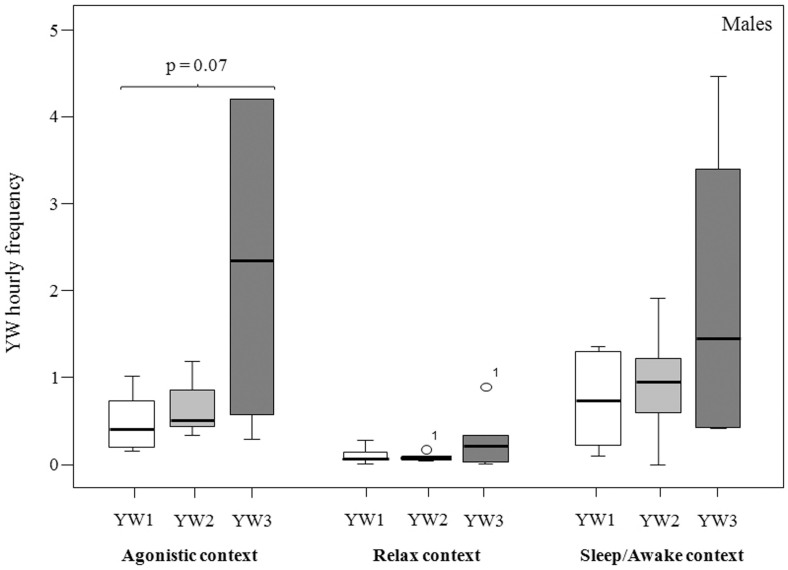
Hourly frequency of male YW1-YW2-YW3 according to the different contexts considered. See the text for the context definition. The box plots show the median and 25th and 75th percentiles; the whiskers indicate the values within 1.5 times the interquartile range, IQR. The open dot indicates an outlier more than 1.5 IQR from the rest of the scores.

**Figure 4:**
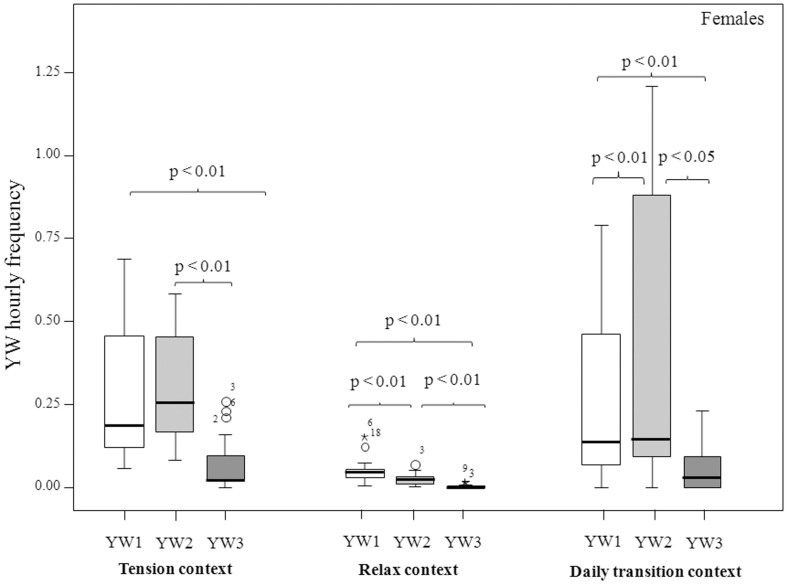
Hourly frequency of female YW1-YW2-YW3 according to the different contexts considered. See the text for the context definition. The box plots show the median and 25th and 75th percentiles; the whiskers indicate the values within 1.5 times the interquartile range, IQR. The open dot indicates an outlier more than 1.5 IQR from the rest of the scores. Asterisks indicate outliers more than 3 IQR from the rest of the scores. Post-hoc analysis (Dunnett's test), only statistical differences are reported: Tension context (YW1-YW3: q = 5.84, p < 0.01; YW2–YW3: q = 5.11, p < 0.01); Relax Context (YW1–YW2: q = 4.09, p < 0.01; YW1–YW3: q = 6.07, p < 0.01; YW2–YW3: q = 4.49, p < 0.01); Daily transition context (YW1–YW2: q = 2.48, p < 0.01; YW1–YW3: q = 3.92, p < 0.01; YW2–YW3: q = 4.53, p < 0.01).

Males and females showed a strong variation in the performance of the three yawn intensities also according to the context (tension, relax, and daily transition; see Methods for context definitions) in which yawning occurred. Under the tension condition, males tended to perform YW3 with a higher frequency compared to the other two yawn types, even though the result failed to reach statistical significance (Friedman's χ^2^ = 5.33, n = 9, df = 2, p = 0.070) (Fig. 3) (Prediction 2b supported for males). Under the conditions of relax and daily transition, the levels of the three types of males' yawns were comparable (relax, Friedman's χ^2^ = 2.33, n = 9, df = 2, p = 0.430; daily transition: Friedman's χ2 = 4.33, n = 9, df = 2, p = 0.311) (Fig. 3). For each context considered, females performed YW1 and YW2 with higher frequencies than YW3 (tension condition: Friedman's χ^2^ = 29.84; n = 20, df = 2, p = 0.0001; relax condition: Friedman's χ^2^ = 37.52, n = 20, df = 2, p = 0.0001; sleep/awake condition: Friedman's χ^2^ = 21.81, n = 20, df = 2, p = 0.0001) (for the results of post-hoc tests see the caption of Fig. 4) (Prediction 2a supported and 2b not supported for females).

As YW1 and YW2 showed a strong similar distribution as a function of sex, rank, and context, we treated them together in the following analyses.

YW3 were more frequently vocalized than YW1&YW2 (Wilcoxon's T = 45; ties = 0; N = 19; p = 0.04) (Prediction 2c supported). We compared the association of lip-smacking (LS) with YW1&YW2 (LS-YW1&YW2), YW3 (LS-YW3), and any affiliative contact (AC: grooming, body contact, play) performed by the subjects (LS-AC). Lip-smacking distribution varied across the three conditions (Friedman's χ^2^ = 25.81, n = 19, df = 2, p = 0.0001). Post-hoc tests revealed that lip smacking was mostly performed in association with the affinitive contacts (LS-AC *vs* LS-YW1&YW2: q = 4.37, p < 0.01; LS-AC *vs* LS-YW3: q = 4.62; p < 0.01) and that lip-smacking was more frequently associated with YW1&YW2 than with YW3 (LS-YW1&YW2 vs LS-YW3: q = 4.86, p < 0.01) (Prediction 2e supported). These analyses were performed on those subjects (n = 19) that showed at least 10 yawns for each of the yawn categories considered (YW1, YW2, YW3).

**Figure 5:**
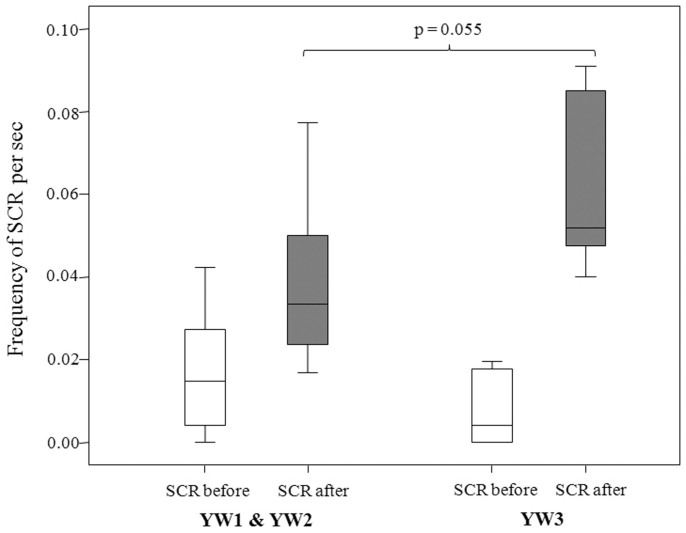
Frequency of scratching (SCR) per second recorded in the 10 sec before and after YW1&YW2 and YW3. The box plots show the median and 25th and 75th percentiles; the whiskers indicate the values within 1.5 times the interquartile range, IQR.

We compared the frequency of scratching (SCR) bouts before and after each yawning event. In order to ascertain that SCR recorded was actually and strictly associated to the yawn event, we restricted our time window to 10 sec, this led to a reduce possibility to intercept a scratching bout. For this reason, we considered only those cases (n = 8) in which at least one SCR was present before and after each yawn. SCR was more frequent in the 10 sec following YW1&YW2 and YW3 than in the 10 sec preceding them (YW1&YW2, Wilcoxon's T = 0; ties = 1; N = 8; p = 0.016; YW3, T = 2, ties = 0, N = 8, p = 0.023). The comparison of SCR bouts recorded during the 10 sec before YW1&YW2 and YW3 did not reveal any significant differences (T = 10.00, ties = 1, N = 8 p = 0.578), whereas SCR tended to be more frequent after YW3 than after YW1&YW2 (T = 4.00, ties = 0, N = 8, p = 0.05; Fig. 5) (Prediction 3 supported).

## Discussion

In this study we found that yawns of different intensity performed by geladas follow a different distribution in frequency depending on the performer, the context, and the behavioural pattern temporally associated to the yawn event.

We investigated the role of social factors on yawn distribution, and what clearly emerged from our data is the relationship between male dominance rank and yawns of higher intensity level (YW3) (Prediction 2a supported; Figure 2), being YW1 and YW2 not affected by the gender of the performer but only by its rank. During YW3 the canine teeth are highly visible and their whitish colour strongly contrasts with the reddish one of the gums and of the internal part of the mouth. This pattern is an evident visual display, which can be easily detected at long distances. Such type of yawn is often accompanied by a loud call preceding the yawn, and/or a long-distance vocalization, thus making it a potential communicative signal easily to be detected at distances that do not require physical proximity. Compared to other baboon species, geladas have larger vocal repertoires. Gustison et al.^52^ found that gelada derived vocalizations are generally used by alpha males while interacting affiliatively with adult females and immediately after intra-OMU fights. The additional auditory component accompanying the most intense form of yawing gives relevance to the visual cue itself (Prediction 2c supported), thus making YW3 a multimodal pattern well perceived by members of the same or different OMUs. Interestingly, male YW3 is frequently displayed during post-conflict and potential competitive periods such as those preceding food distribution (Prediction 2b supported) while, in similar contexts, females showed high levels of YW1 and YW2. This suggests that either YW3 has different functions in males and females or that it is so rare in females that it can be considered primarily a male signal. Moreover, it is worth noting that a vocalized yawn performed by the alpha male of one of the two OMUs often elicited the same response in the alpha male of the other OMU, thus indicating a potential function of inter-group communication between males that are potential rivals. Based on our findings, we hypothesize that YW3 can be used by high ranking males as a multimodal display with the function of intimidating conspecifics especially during situations characterized by high levels of social tension (Figure 3). Even though, to fully understand the communicative function of a behaviour, the response of potential receivers has to be investigated before a display could be labelled as a signal.

Yet, the hypothesis of the threatening function of YW3 in geladas finds support in the literature. In some nonhuman primates with evident sexual dimorphism, males yawn considerably more frequently than females do^10^,^11^,^20^,^41^,^53^, even though no distinction has been made between different types of yawn patterns. Moreover, in long-tailed macaque males Chambers and Phoenix^54^ found a positive correlation between testosterone levels and rates of spontaneous yawning, which was mainly associated with inter-male threats^12^. The display function of yawning in dimorphic species is also supported by the absence of sex difference in yawn frequency in those primate species characterized by a low level of sexual dimorphism in canine size, such as humans (*Homo sapiens*^7^,^45^), chimpanzees (*Pan troglodytes*^14^), and lemurs (*Lemur catta* and *Propithecus verrauxi*, Palagi et al. unpublished data). Even though some social factors appear to modulate the use of yawn as a threatening signal in geladas, it could be interesting to investigate the potential link between the different types of yawning and androgen hormone concentration (e.g. testosterone).

Even though LMM revealed that YW1 and YW2 were only influenced by dominance rank (Prediction 2d supported), females preferentially performed YW1 and YW2 in each of the contexts considered for the analyses (Figure 4). The association of lip-smacking (LS) with YW1 and YW2 did not reach the baseline level of LS (measured by the association of LS with the main affiliative interactions^36^,^55^). Yet, YW1 and YW2 were more frequently associated with LS compared to YW3. It seems that, contrary to YW3, the less intense forms of yawning are more linked to positive social contexts thus adding a further element of dichotomy between the different yawn intensities considered (Prediction 2e supported). Hence, compared to YW3, YW1 and YW2 can be read as an expression of benign intent by high ranking individuals towards other group members, especially females. Along with previous findings on yawn contagion in this species^13^, these data suggest that YW1 and YW2 are commonly used by gelada females as part of a complex communicative system among individuals that often engage in affiliative interactions and that are emotionally connected. In fact, the relationships within the typical gelada one-male unit (OMU) revolve around adult females, who form the core of the cohesion and stability typical of OMUs^56^. In some cases, the strength of female bonds suffices to maintain OMU integrity despite the absence of the male^28^. It is worth noting that between gelada females only YW1 and YW2 elicited a precise mirroring during yawn contagion^13^ thus suggesting the importance of these two types of yawn as strong stimuli in triggering a matched response (YW1/YW1 and YW2/YW2). The high frequency and accuracy of contagiousness elicited by YW1 and YW2 can have not only important implication in synchronizing the activity between individuals, but it may also strengthen their bonds and, at the same time, signal their relationship quality^13^.

The arousal hypothesis of yawning predicts that it can be considered as a displacement behaviour associated with neural mechanisms lowering the arousal level in the subject (humans^12^; mangabeys and macaques^10^; macaques^21^,^57^; hamadryads^56^). In the first gelada's ethogramm, yawning - similarly to scratching - was described as a self-directed behaviour indicating anxiety^28^. Our data show that scratching increased after each yawning type, even though animals tended to scratch themselves more after yawning of higher intensity level (YW3), thus suggesting that YW3 could indicate an even higher level of arousal (Figure 5).

As it occurs in other primate species, in which yawning shows predictable daily variations^1^,^7^,^8^,^10^,^18^,^28^,^58^, in geladas we found that animals yawned preferentially in the phase of sleep/awake transition, especially in the early mornings (Prediction 3 partially supported). Yet, we detected a difference in the yawn temporal distribution according to the spectrum intensity of yawns considered. In early morning, the first type of yawn to increase was YW1, which peaked from 07.00 to 09.00 am. During this time window the subjects alternated short periods of sleeping and waking, being social activities (e.g. grooming, care giving) not already begun. YW2 and YW3 peaked an hour later, from 08.00 to 09.00 am. During this period animals were completely awake and all engaged in their usual social activities. We could tentatively consider the early morning YW1 (the yawn of the lowest intensity) as a form of yawn which, by promoting full vigilance and awakening, favours the beginning of group-coordinated activities (e.g. feeding, grooming). To date, there are no studies investigating the relation between the daily distribution of yawning and its different intensity or duration in time. Hence, it is not yet possible to compare our findings with other reports and, therefore, our hypothesis remains speculative and needs further investigation.

The only type of yawn showing a second peak along the day was YW3, which was particularly frequent from 02.00 to 03.00 pm. This time window included the afternoon pre-feeding, a period characterized by high levels of anxiety and aggressive events. As already stated, compared to YW1 and YW2, YW3 was more frequently associated with social tension situations and, under such circumstances, it was mainly used by males as a threatening signal. Baenninger^4^ reported that lions (*Panthera leo*) and mandrills (*Papio sphinx*) yawned more frequently just before feeding time, a finding that was replicated by Holmgren et al.^59^ in laboratory rats, even though no distinction in the morphology of the yawn was reported.

In conclusion, even though the functional distinction of the different intensities of gelada yawns is not so clear-cut, our data seem to indicate that yawns of different intensity have multiple communicative functions (e.g., synchronization of group activity, emotional connection, inter-group communication and threatening). Our findings also suggest that in geladas the spectrum of yawn intensity varies according to the sex of the yawner, with a strong dimorphism which can reflect, at least in part, on the potential communicative functions of yawning.

## Methods

### Subjects and housing

The colony of geladas, housed at the NaturZoo (Rheine, Germany), is held in two enclosures with both an indoor (a room about 36 m^2^) and outdoor facility (an island of 2,700 m^2^ surrounded by a boundary ditch). The size of the island allows the scattering of geladas and, consequently, the formation of small groups of animals that frequently change. The enclosures are equipped with everything necessary to allow the geladas to move freely in all three dimensions. Specifically, the outside enclosure is located in an open naturally hilly area equipped with trees, branches, ropes, and dens. The colony of geladas was made up of two one-male units (OMUs) (for group's composition and age-class definition see Table 1). Individual identification was based on sex, age, and distinctive external features (scars, size, missing fur patches, fur colour, and facial traits). Kinship between animals was known. In 2007, the two OMUs were housed in the same enclosure and, in 2009–2010, they lived separately in two different enclosures. The animals are fed with grass, vegetables, and pellets, which are scattered on the ground twice a day (9:30 a.m., 2:30 p.m.). Water is available *ad libitum*. No stereotypic or aberrant behaviours have been observed in this group.

This study was approved by the University of Pisa (Animal Care and Use board). Since the study was purely observational the committee waived the need for a permit.

### Data collection

We collected behavioural data during a 4-month period in 2007 (June–September), a 4-month period in 2009 (June–September), a 2-month period in 2010 (July–August) and a 2-month period in 2011 (July–August). Data were collected through a voice and a video recorder. We gathered 1,809 hours of observations, which took place daily over 6-hr periods that we spanned morning (from 6:00 a.m.) and evening (until 9:00 p.m.). In 2007 we collected data from 6:00 am to 10:00 pm in order to analyse the daily yawn distribution.

Data were collected by two observers, who were trained by the same person (E.P.). The presence of two observers was necessary for the concurrent use of the diverse techniques of observation. Training was over when the observations produced a Cohen's kappa higher than 0.70. We checked for observation reliability at the beginning of each month obtaining values never below 0.70.

### Operational definitions and data analyses

By using *all-occurrences sampling*^60^ we collected and video-recorded the yawning events performed by all subjects (Table 1).

During each yawning occurrence we recorded the exact time of the yawn, the posture assumed by the yawner (defined as sitting, standing, and lying), the identity of the yawner, and the types of yawn animals performed (YW1, YW2, YW3). Since in geladas, each type of yawning display can be accompanied by a loud precall and/or a long-distance vocalization^28^, for each yawn event we recorded both the visual and auditory modality. Moreover, we sampled the behavioural item occurring during the 10 s before and 10 s after the yawn^10^) that included further ethogram items. Specifically, we recorded a series of state of activity and behaviours classified into social and non social. Resting, sleeping, walking, standing, grooming, feeding, contact sitting, and proximity were scored as states. Other behavioural items, such as raised eye brows, scratching, self-grooming, body shake, lip flip, urination, defecation, gravel digging, and copulation were scored as events. For each behavioural item we recorded the identity of the actor and the receiver.

To calculate the rate of vocalized yawns, we counted how many times a yawn of a given intensity (e.g., YW3) was associated with a loud precall and/or a long-distance vocalization (e.g., vocalized YW3) and divided the number obtained on the total amount of yawns recorded for such intensity (total of YW3).

Scratching was recorded as a behavioural measure of anxiety experienced by the subject^21^,^27^,^29^,^49^,^50^. We defined scratching as a repeated movement of the hand or foot during which the fingertips are drawn across the individual's fur. A new scratching bout was assigned when the scratched body part changed.

We determined the ranking position of each subject that was assessed for each observation period (2007–2009–2010–2011) by entering decided conflicts into a winner/loser socio-matrix. Such socio-matrices were reordered via Matman 1.0 and three rank levels were recognized: high (if an animal's rank fell into the upper rank quartile), low (if animal's rank fell into the lower rank quartile), and medium (if an animal's rank fell between the lower and the upper quartile).

We defined three different contexts during which yawning could occur: tension, relax, and daily transition. The tension context included prefeeding time (30 min before food distribution: 9:00–9:30 a.m. and 2:00–2:30 p.m.), feeding time (9.30–10:00 a.m. and 2:30–3:00 p.m.) and post-conflict periods (10 min after the end of an agonistic encounter). By preliminary observations we were able to identify the periods of daily transition of geladas. The daily transition context was characterized by a behavioural change from inactivity to activity (early morning; 06.00–8:30 a.m.) and from activity to inactivity (late evening; 8:00–10:00 p.m.). The relax context included all those periods during which the animals were active and engaged in social interactions like play, grooming, contact sitting, infant care, resting, and environment exploration (10:00 a.m.–02:00 p.m. and 03:00–08:00 p.m.).

Due to non-normality of data we applied non-parametric statistics (Mann-Whitney U test; Wilcoxon Matched-Pairs Signed Rank test; Friedman's Two-way Anova for ranks test).

By using Linear Mixed Models (LMM) we examined the effect of sex and rank on each of the types of yawn performed (YW1, YW2, YW3). Rank and sex were entered as fixed variables; while the yawner identity and observational periods were entered as random factors (nominal variables) (Table 2). We tested the model for the variables of interest, spanning from a single-variable model to a model including all the fixed factors (full model). To select the best model, we used the Akaike's Corrected Information Criterion (AICc), a measure for comparing mixed models based on the -2 (Restricted) log likelihood. The AICc corrects the Akaike's Information Criterion (AIC) for small sample sizes. As the sample size increases, the AICc converges to AIC. The model with a lower value of AIC was considered to be the best model.

## Figures and Tables

**Table 1:** The group of geladas (*Theropithecus gelada*) housed in the NaturZoo (Rheine, Germany). The One-Male Units (OMUs) are indicated

Subjects	Year of Birth	Mother	Sex class	Age class	Observational period
Gerda (OMU1)*	1978	Unknown	F	Adult: > 6 years	2007
Gertje (OMU1)	1987	Gerda	F	2007–2011	
Gitta (OMU1)	1992	Gertje	F	2007–2011	
Albert (OMU1)*	1993	Agathe	M	2007	
Amadeus (OMU2)*	1994	Afra	M	2007	
Gloria (OMU1)	1994	Gertje	F	2007–2011	
Gevia (OMU1)	1996	Gitta	F	2007–2011	
Gwladys (OMU2)*	1997	Gesa	F	2007	
Günni (OMU2)*	1997	Gertje	F	2007	
Hilfia (OMU1)	2001	Gevia	F	2007–2011	
Angel (OMU3)	1996	Agathe	F	2009–2011	
Alegria (OMU3)	1998	Aurora	F	2009–2011	
Adina (OMU3)	1998	Agathe	M	2009–2011	
Dominick (OMU1)	2001	Buffy	F	2009–2011	
Hilfia (OMU1)	2001	Gevia	F	2009–2011	
Bangle (OMU3)	2002	Angel	F	2009–2011	
Bako (OMU3)	2003	Sereba	M	2009–2011	
Babs (OMU3)	2003	Alegria	F	2009–2011	
Heike (OMU1)	2003	Gloria	F	2007–2011	
Hera (OMU1)	2003	Grace	F	2007–2011	
Helena (OMU1)	2004	Gitta	F	2007–2011	
Hector (OMU1)*	2002	Gitta	M	Sub-adult: 4.5–6 years	2007
Hobbit (OMU1)*	2002	Gloria	M	2007	
Jacques (OMU2)*	2003	Gwladys	M	2007	
Hagos (OMU1)	2005	Gloria	M	2007–2011	
Bern (OMU3)	2005	Adina	M	2009–2011	
Hermine (OMU1)	2005	Gitta	F	2007–2011	
Bounty (OMU3)	2005	Alegria	F	2009–2011	
Belinda (OMU3)	2005	Angel	F	2009–2011	
Herkules (OMU1)*	2003	Gevia	M	Juvenile: 2.5–4.5 years	2007
Hichele (OMU1)	2007	Gevia	M	2007–2011	
Jasper (OMU2)*	2005	Gwladys	M	Infant: 6 months–2.5 years	2007
Tommaso (OMU3)	2009	Adina	M	2009–2011	
Giada (OMU3)	2009	Alegria	F	2009–2011	
Alessia (OMU3)	2009	Babs	F	2009–2011	
Betta (OMU1)	2009	Gitta	F	2009–2011	
Davide (OMU3)	2009	Angel	M	2009–2011	
Dusella (OMU1)	2009	Helena	F	2009–2011	
Dalia (OMU1)	2009	Gloria	F	2009–2011	
Dita (OMU1)	2009–2010	Hera	F	2009–2011	
Debi (OMU1)	2009	Grigia	F	2009–2011	
Diana (OMU1)	2010	Hilfia	F	2010–2011	
Che (OMU1)	2010	Günni	F	2010–2011	
Giulia (OMU3)	2010	Adina	F	2010–2011	
Filippa (OMU3)	2010	Alegria	F	2010–2011	
Gaga (OMU1)	2010	Hermine	F	2010–2011	
Alexandra (OMU3)	2010	Belinda	F	2010–2011	
Julie (OMU1)*	2007	Günni	F	Black-Infant: 1–6 months	2007
Ar (OMU1)	2011	Hera	M	2011	

**Note:** The asterisk (*) indicates the subjects who were removed from the zoo after 2007.

**Table 2:** Description of variables used in LMM analyses

The dependent variables tested separately	Type of variable
YW1	Scale
YW2	Scale
YW3	Scale
Fixed Explanatory variables	
Rank	Ordinal (1 = high, 2 = medium, 3 = low)
Sex	Dichotomous (1 = female, 0 = male)
Random variables	
Yawner's identity	Nominal
Observational Period	Ordinal (1 = 2007; 2 = 2009; 3 = 2010; 4 = 2011)

**Table 3:** Best LMM explaining the frequency of triadic affiliation as a function of the relationship quality between victim and bystander

**YW1** (AICc = −142.760)	**Co**	**SE**	**t**	**p**	**CI (95%) Lower-Upper**
**Fixed Explanatory Variables**					
Intercept	0.125	0.031	4.020	0.010	(0.045)–(0.205)
Rank 0	−0.078	0.021	−3.606	0.001	(−0.121)–(−0.035)
Rank 1	−0.060	0.017	−3.529	0.001	(−0.095)–(−0.026)
Rank 2	0^a^	0			
Sex 0	−0.003	0.019	−0.153	0.879	(−0.040)–(0.035)
Sex 1	0^a^	0			
**Random variables**	**Variance**	**SE**			
Yawner identity	0.0000^a^	0			
Period	864.063	732588			
**YW2** (AICc = −160.549)	**Co**	**SE**	**t**	**p**	**CI (95%) Lower-Upper**
**Fixed Explanatory Variables**					
Intercept	0.099	0.028	3.515	0.020	(0.025)–(0.175)
Rank 0	−0.059	0.018	−3.258	0.003	(−0.096)–(−0.022)
Rank 1	−0.042	0.014	−2.948	0.006	(−0.071)–(−0.013)
Rank 2	0^a^	0			
Sex 0	0.029	0.016	1.885	0.069	(−0.002)–(0.061)
Sex 1	0^a^	0			
**Random variables**	**Variance**	**SE**			
Yawner identity	0.000005	0.0003			
Period	0.002	0.002			
**YW3 (AICc = −11.430)**	**Co**	**SE**	**t**	**p**	**CI (95%) Lower-Upper**
**Fixed Explanatory Variables**					
Intercept	0.145	0.060	2.407	0.024	(0.020)–(0.269)
Rank 0	−0.183	0.081	−2.255	0.028	(−0.347)–(−0.020)
Rank 1	−0.162	0.063	−2.570	0.013	(−0.290)–(−0.036)
Rank 2	0^a^	0			
Sex 0	0.320	0.068	4.664	0.0001	(0.182)–(0.458)
Sex 1	0^a^	0			
**Random variables**	**Variance**	**SE**			
Yawner identity	0.000001	0.0000001			
Period	0.001	0.003			

Co: coefficient; SE: standard error; CI (95%): Confidence Interval;

aThis coefficient is redundant.
